# Impact of providing walnut samples in a lifestyle intervention for weight loss: a secondary analysis of the HealthTrack trial

**DOI:** 10.1080/16546628.2017.1344522

**Published:** 2017-07-03

**Authors:** Elizabeth P. Neale, Linda C. Tapsell, Allison Martin, Marijka J. Batterham, Cinthya Wibisono, Yasmine C. Probst

**Affiliations:** ^a^ School of Medicine, Faculty of Science, Medicine and Health, University of Wollongong, Wollongong, Australia; ^b^ Illawarra Health and Medical Research Institute, University of Wollongong, Wollongong, Australia; ^c^ Statistical Consulting Service, School of Mathematics and Applied Statistics, Faculty of Engineering and Information Sciences, University of Wollongong, Wollongong, Australia

**Keywords:** Walnut, weight loss, diet quality, discretionary foods

## Abstract

**Background**: Being more specific about individual food choices may be advantageous for weight loss. Including a healthy food (e.g. walnuts) may help to expose effects.

**Objective**: To examine the impact of including walnuts in diets for weight loss.

**Design**: Secondary analysis of the HealthTrack lifestyle intervention trial. Overweight and obese participants were randomized to: usual care (*C*), interdisciplinary intervention including individualized dietary advice (*I*), or interdisciplinary intervention including 30 g walnuts/day (*IW*). Changes in body weight, energy intake, intake of key foods, physical activity, and mental health over three and 12 months were explored.

**Results**: A total of 293 participants completed the intensive three-month study period, and 175 had data available at 12 months. The *IW* group achieved the greatest weight loss at three months. *IW* reported significant improvements in healthy food choices, and decreased intakes of discretionary foods/beverages, compared to *C*. Weight loss remained greatest in *IW* at 12 months.

**Discussion**: Significant effects were seen after three months, with the *IW* group achieving greater weight loss and more favorable changes in food choices.

**Conclusions**: Including 30 grams walnuts/day in an individualized diet produced weight loss and positive changes in food choice.

## Introduction

In the clinical setting, lifestyle interventions that focus on dietary, physical activity, and psychological components are the most effective in promoting weight loss [1]. There is substantial evidence for the effects of different types of food on metabolic parameters; therefore giving specific advice on foods choices may enhance such effects. Translating dietary guidelines into food choices that suit an individual’s usual eating pattern may be more helpful than general advice. For experimental purposes, providing a sample of a healthy food may further expose the impact of this translational effort.

From the literature, nuts could be seen as an exemplary food. The habitual consumption of tree nuts has been associated with reduced coronary heart disease risk [2–5], and walnuts in particular, with their unique nutrient content may enhance these effects. In the first instance, with a high proportion of polyunsaturated fatty acids (including alpha-linolenic acid, ALA), walnuts can improve overall dietary fatty acid profile [6] and, like many tree nuts, deliver dietary phytosterols and fibre [7]. Clinical trials have shown that consuming walnuts can lead to improvements in lipid profiles [8] and favorable changes in endothelial function [9,10].

Importantly, studies exploring habitual consumption of a limited amount of nuts (for example approximately 56 grams or more per week [11], or 28 grams per day [12]) have not found associations with weight gain, despite nuts being energy-dense and high in fat [11–13]. In fact, not all of the energy may be available, as recent research shows that the conventionally applied energy value for walnuts is an over-estimation by more than 20% [14]. Energy balance and body weight are, however, the product of a total diet, so the dietary context in which walnuts are consumed is relevant. At a food group level, population surveys suggest that nut consumers may also have higher intakes of other healthy foods such as fruits and dark-green vegetables compared to non-consumers of nuts [15,16]. Baseline analysis from the PREDIMED trial revealed that frequent consumption of nuts was associated with a significantly higher reported intake of fruit, vegetables, and fish [17]. In a smaller clinical trial, the regular provision of walnuts resulted in improvements in overall diet quality [18]. Further research is now needed to explore how this may occur, whereby including walnuts in the diet may influence intakes of other key foods by dietary association, resulting in positive health effects.

The aim of this analysis was to examine the impact of regular walnut consumption on weight loss, energy intake, and the consumption of key foods in the context of a lifestyle intervention trial targeting weight loss. A per-protocol analysis was conducted to ensure the results reflected adherence with the prescribed walnut intake. This analysis considered effects during both the intensive phase of the study (three months), as well as the longer-term follow-up (12 months).

## Materials and methods

### Healthtrack study context

This was a secondary analysis of the HealthTrack study, a 12-month randomized controlled trial that tested the effect of an interdisciplinary intervention on weight loss in overweight and obese adults [19]. An intensive phase was conducted for three months (monthly clinic visits), followed by quarterly follow up visits to 12 months.

Participants were eligible to take part in the study if they were permanent residents of the Illawarra region of Australia, with a body mass index (BMI) of 25–40 kg/m^2^. Participants were screened for nut allergy in order to be eligible to participate in the study. Participants were randomized to receive either general dietary advice (usual care, *C*), interdisciplinary intervention including individualized dietary advice, (*I*), or interdisciplinary intervention plus 30g walnuts/day (*IW*).

All groups received dietary advice based on the food groups forming the Australian Guide to Healthy Eating (AGHE) [20], namely vegetables, fruit, cereals/grains, lean meat and alternatives (including fish and seafood), and low fat dairy foods. The *C* group was given general advice from a practice nurse with reference to standard servings from AGHE related pamphlets, as well as receiving National Physical Activity Guidelines [21]. The diet plans for participants in the *I* and *IW* groups were individualized with a prescribed number of serves of each food group to meet energy intake targets, and the dietary advice was delivered by Accredited Practising Dietitians (APDs). For the *IW* group, the diet plan included the free sample of 30g walnuts/day provided for the duration of the study. The energy value of the walnuts was modelled into the overall diet plan. The advice was accompanied by menu-style suggestions. Consultations with APDs (*I* and *IW* groups) also included categorical exercise advice, again following the National Physical Activity Guidelines and supported by an exercise physiologist if requested. Participants in both intervention groups also received quarterly phone calls from a trained health coach, who counselled participants on Acceptance and Commitment therapy via a printed workbook.

Ethics approval was granted by the University of Wollongong/Illawarra Shoalhaven Local Health District Human Research Ethics Committee (Health and Medical) (HE 13/189). All participants gave written informed consent to participate in the study. The study was registered with the Australian and New Zealand Clinical Trial Registry (ANZCTRN 12614000581662).

Body weight (kg) was measured at baseline, three, and 12 months with participants in an upright position, with minimal clothing and no shoes (Tanita TBF-662, Wedderburn Pty Ltd, Ingleburn, NSW, Australia). Physical activity (MET-mins/week) was determined using the International Physical Activity Questionnaire (IPAQ) short form [22]. Psychological measures included the Depression Anxiety Stress Scale (DASS-21), and the SF12 physical and mental component summaries (quality of life) [23]. At clinic visits, participants randomized to the *IW* group reported the number of days they did not consume the provided walnuts in the previous month.

Dietary intakes for all participants was assessed by a different APD to the one providing the dietary advice, using a validated protocol for diet history interviews [24]. All dietary data was analyzed using Foodworks software (Version 7, Xyris Software, QLD, Australia, 2012), using the AUSNUT 2007 food composition database [25], the most up-to-date food composition database at the time of study commencement. In order to utilize the most recent food group classification system, which follows a nested hierarchical structure of major, sub-major and minor groups, dietary data was subsequently updated to AUSNUT 2011–13[26] via a systematic process described elsewhere [27].

This study also sought to explore intakes of energy from two different types of foods, those being nutrient-rich or ‘core’ foods, and discretionary foods/beverages, defined as those foods not essential for a healthy diet and which tend to be high in saturated fat, and/or added salt, added sugars, or alcohol [20]. The classification of foods as ‘discretionary’ or ‘core’ was conducted using the food lists associated with the National Nutrition and Physical Activity Survey (NNPAS) [28]. Specifically, energy provided by core foods was calculated for foods in the AUSNUT 2011–13 categories of *fruit products and dishes, vegetable products and dishes*, and *fish and seafood products and dishes*. These food groups were selected based on research suggesting different consumption patterns between those who regularly consume nuts and non-nut consumers [15–17]. Energy provided by discretionary foods/beverages was also determined.

### Measurement of walnut intake

Adherence to the walnut intervention was calculated based on the number of days participants reported consuming walnuts (30g/day, provided in single serve packages) in the previous month. A total percentage adherence rate over the duration of their involvement in the study was calculated for each participant.

For all groups, the consumption of nuts in general and walnuts in particular was identified from diet history interview data. The daily consumption (grams) of all nuts and of walnuts was calculated. In the case of mixed products such as trail mixes, the percentage amount of nuts were calculated based on the AUSNUT 2007 recipe file [29]. Products such as muesli and muesli bars were not included in the current analysis due to the lack of consistent reporting of percentage amount of nuts in different products.

### Statistical analysis

Statistical analysis was conducted using SPSS (version 21.0, IBM Corp, USA, 2012). Median percentage adherence to walnut provision in the *IW* group was determined. Consumption of total nuts and walnuts as reported during diet history interviews was then calculated at baseline, three, and 12 months for all study participants. Within and between-group comparisons for total nut and walnut intake were conducted using Friedman tests and Kruskal-Wallis tests, respectively, with Bonferroni adjustment for post-hoc tests.

In accordance with previous research [30,31], an acceptable level of adherence to the walnut prescription was classified as self-reported consumption ≥ 80% of the provided walnut dose, i.e. ≥ 24 grams per day. For subsequent analyses, participants in the *IW* group who reported consuming less than 24 grams/day were excluded from further analyses.

Changes in body weight, total energy intake, energy intake from key AUSNUT 2011–13 major food groups (*fruit products and dishes, vegetable products and dishes*, and *fish and seafood products and dishes*), energy intake from discretionary foods/beverages, self-reported physical activity (MET-mins/week), DASS-21, and SF12 physical and mental component summaries were compared between groups using a Kruskal-Wallis test due to the non-parametric distribution of the data; assumption violations led us to choose this approach over ANCOVA [32]. Significant differences were explored using post-hoc Mann-Whitney tests with Bonferroni adjustment. As an indicator of dietary behaviors overall, percentage energy intake from discretionary foods/beverages was also calculated for baseline, three months, and 12 months. Within and between-group comparisons were conducted using Friedman tests and Kruskal-Wallis tests, respectively, with Bonferroni adjustment for post-hoc tests. Statistical significant was set at *P *< 0.05.

## Results

### Participant characteristics

**Figure 1.**
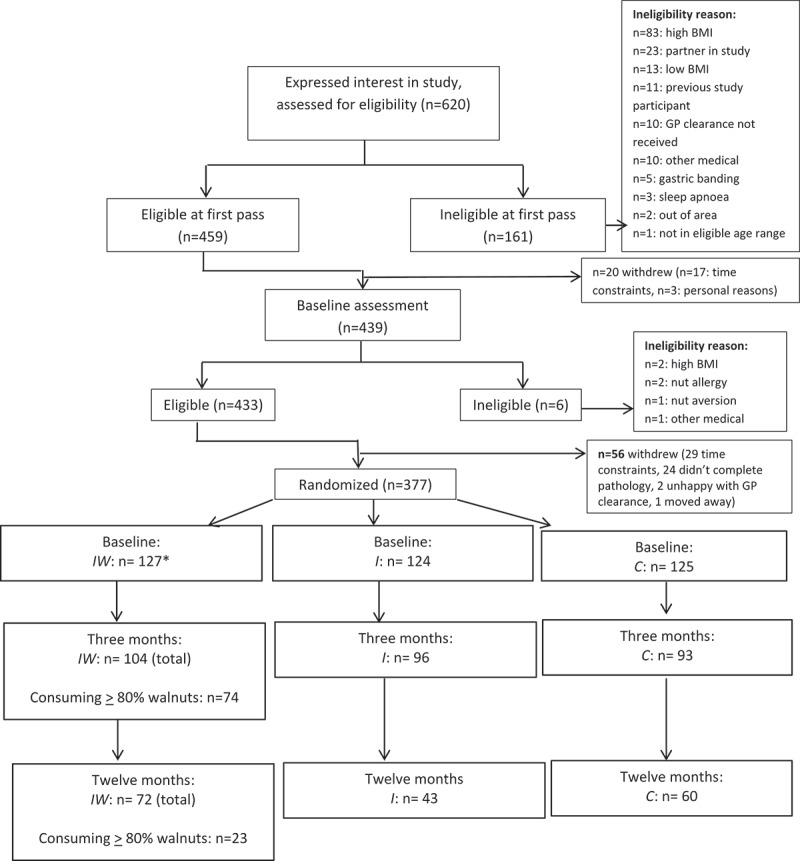
Participant flow in the HealthTrack randomized controlled trial *n = 1 was randomized to *I* but provided with walnuts, therefore was treated as *IW* for this analysis.

A total of 377 participants were randomized to the HealthTrack study (Figure 1). Table 1 provides a summary of baseline characteristics. As this was a per protocol analysis, one participant who was randomized to *I* but provided with walnuts was treated as *IW* for subsequent analyses. A total of 293 participants completed the intensive three-month period of the intervention, and n = 175 had dietary data available at 12 months.

**Table 1.** Baseline characteristics of participants in the HealthTrack study.

	Control group	Intervention group	Intervention + walnut group
n	126	124	127^
Age (years)*	43.80 ± 7.46	43.79 ± 7.97	42.10 ± 8.69
Gender (% female)	73%	73%	75%
Body weight (kg)*	91.84 ± 14.69	91.86 ± 15.22	91.38 ± 15.51
BMI (kg/m^2^)*	32.49 ± 4.12	32.59 ± 4.25	32.63 ± 4.28
kJ intake†	9400 (7840–11 574)	8666 (7176–11 004)	8810 (7445–10 783)
Fish and seafood products and dishes (kJ) †	122 (5–287)	166 (17–328)	128 (0–271)
Fruit products and dishes (kJ) †	364 (164–602)	315 (160–584)	286 (135–490)
Vegetable products and dishes (kJ) †	520 (353–848)	519 (348–762)	540 (387–831)
Discretionary foods and beverages (kJ) †	2543 (1814–3931)	2465 (1493–4020)	2685 (1799–3898)
MET-mins/week†‡	876 (396–1523)	921 (392–1552)	1040 (568–2328)
DASS-21†	13 (6–19)	11 (7–19)	11 (7–18)
SF-12 Physical component score†§	49 (43–54)	50 (45–54)	51 (45–55)
SF-12 Mental component score†	49 (41–53)	48 (38–55)	48 (39–54)

^n = 1 participant randomized to *I* was provided with walnuts, they were treated as *IW* for this per-protocol analysis

*Values are mean ± standard deviation

†Values are median (interquartile range)

‡Baseline sample size: control (n = 124), intervention (n = 122), intervention + walnut (n = 125)

§ Baseline sample size: control (n = 126), intervention (n = 123), intervention + walnut (n = 126)

### Walnut consumption

The median (interquartile range) walnut adherence rate in the *IW* group over the duration of the study was 85.33% (66.53–94.75).

There were no significant differences between groups in self-reported total nut or walnut consumption at baseline (Table 2). Post-hoc tests indicated that participants in the *IW* group reported significantly increased intakes of total nuts and walnuts over the first three months of the study (both *P *< 0.001). Reported consumption of both total nuts and walnuts decreased between three and 12 months in the *IW* group (total nuts: *P *= 0.003, walnuts: *P *< 0.001), but remained significantly higher at 12 months than at baseline (total nuts: *P *= 0.001, walnuts: *P *< 0.001). Participants in the *I* and *C* groups did not report changing their intake of total nuts or walnuts over time.

**Table 2.** Median (IQR) total nut and walnut intake per day (grams) as reported in diet history interviews.

		Control		Intervention		Intervention + walnuts	
Variable	*n*	Value	*n*	Value	*n*	value	p-value (between groups)†
**Total nut intake, median (IQR), grams/day**							
Baseline	126	7.1 (0.0–20.6)	124	6.1 (0.0–15.0)	127	4.9 (0.0–17.9)^x^	0.662
Three months	93	6.8 (0.0–20.6)^a^	96	2.7 (0.0–8.9)^a^	104	30.0 (25.7–31.3)^b, y^	**0.000**
12 months	60	8.6 (2.2–17.9)^a^	43	7.1 (0.0–15.0)^a^	72	23.4 (8.6–30.0)^b, z^	**0.000**
**p-value (time)‡**		0.385		0.191		**0.000**	
**Walnut intake, median (IQR), grams/day**							
Baseline	126	0.0 (0.0–0.0)	124	0.0 (0.0–0.0)	127	0.0 (0.0–0.0)^x^	0.389
Three months	93	0.0 (0.0–0.0)^a^	96	0.0 (0.0–0.0)^a^	104	30.0 (22.0–30.0)^b, y^	**0.000**
12 months	60	0.0 (0.0–0.0)^a^	43	0.0 (0.0–0.0)^a^	72	17.1 (4.3–30.0)^b, z^	**0.000**
**p-value (time)‡**		0.209		0.761		**0.000**	

Superscripts indicate significant differences between groups (a, b) and within groups (x, y, z) after Bonferonni adjustment

**†** Kruskal-Wallis test

**‡** Friedman test

### Changes in body weight, dietary variables, physical activity, and mental health

At three months, 74/104 (71%) participants in the *IW* group reported consuming ≥ 80% of the provided walnuts. Data from these participants were included in subsequent analyses. By 12 months, only 23/72 (32%) of the *IW* participants achieved an acceptable level of adherence. This means that, of the retained participants, about 30% did not fully consume the samples and this proportion doubled at 12 months.

Over the first three months of the study, significant differences in weight loss were observed between study groups (Table 3). Post-hoc tests indicated significantly greater weight loss was reported in the *IW* group compared to *C* (*P *= 0.012). Over 12 months, the *IW* group achieved the greatest weight loss, and weight loss was significantly greater in the *IW* group than *C* (*P *= 0.028) (Table 4). There were no significant differences in weight loss detected between the *IW* and *I* groups at either three or 12 months, nor between the *I* and *C* groups.

**Table 3.** Median (interquartile range) change over three months between study groups.

Variable	Control group (n = 93)	Intervention group (n = 96)	Intervention + walnut group (n = 74)*	P-value
Body weight (kg)	−1.60 (−2.50 – −0.10)^a^	−2.00 (−4.00 – −0.35)^a, b^	−2.45 (−5.23 – −0.48)^b^	0.011
Energy (kJ)	−1752 (−3469 – −45)	−2047 (−3769 – −640)	−1591 (−2637 – −641)	0.295
Fish and seafood products and dishes (kJ)	3 (−87 – 166)	2 (−70 – 177)	87 (−27 – 232)	0.162
Fruit products and dishes (kJ)	52 (−93 – 238)^a^	125 (−180 – 382)^a, b^	196 (−29 – 355)^b^	0.042
Vegetable products and dishes (kJ)	19 (−310 – 275)	24 (−159 – 268)	56 (−231 – 383)	0.544
Discretionary foods and beverages (kJ)	−758 (−1982 – −159)^a^	−1155 (−2654 – −346)^a, b^	−1457 (−2441 – −606)^b^	0.020
MET-mins/week†	489 (−196.5 – 1457)	401 (−150 – 1257)	1095 (198 – 1846)	0.083
DASS-21‡	−1 (−5–2)	−1 (−6 – 2)	−1 (−6 – 1)	0.667
SF12 Physical component summary§	1 (−2 – 5)	0 (−3 – 3)	2 (−2 – 6)	0.639
SF12 Mental component summary§	2 (−1 – 7)	1 (−4 – 7)	2 (−2 – 8)	0.094

Superscripts indicate significant differences between groups (a, b) after Bonferroni adjustment

*Analysis restricted to Intervention + walnut participants who consumed at least 80% of the provided walnuts

† Available sample size: control (n = 90), intervention (n = 91), intervention + walnut (n = 71)

‡ Available sample size: control (n = 92), intervention (n = 93), intervention + walnut (n = 74)

§ Available sample size: control (n = 91), intervention (n = 94), intervention + walnut (n = 72)

**Table 4.** Median (interquartile range) change over 12 months between study groups.

Variable	Control group (n = 60)	Intervention group (n = 43)	Intervention + walnut group (n = 23)*	P-value
Body weight (kg)	−1.10 (−4.18 – 0.50)^a^	−2.40 (−7.70 – 0.90)^a, b^	−4.60 (−10.70 – −1.20)^b^	0.026
Energy (kJ)	−1508 (−3115 – 137)	−1710 (−3279 – −210)	−561 (−1988 – 651)	0.111
Fish and seafood products and dishes (kJ)	0 (−158 – 83)	0 (−92 – 96)	0 (−156 – 433)	0.759
Fruit products and dishes (kJ)	12 (−223 – 126)	22 (−327 – 209)	71 (−101 – 304)	0.526
Vegetable products and dishes (kJ)	−10 (−257 – 251)	−19 (−250 – 203)	−38 (−526 – 330)	1.000
Discretionary foods and beverages (kJ)	−647 (−2392 – 212)	−943 (−2025 – −56)	−608 (−1358 – 270)	0.624
MET-mins/week†	840 (−80 – 2247)	548 (−68 – 1643)	948 (−238 – 3095)	0.696
DASS-21‡	−2 (−5 – 1)	−1 (−6 – 4)	−1 (−6 – 2)	0.869
SF12 Physical component summary§	3 (−1 – 8)	2 (−1 – 5)	2 (−2 – 9)	0.526
SF12 Mental component summary§	3 (−3 – 7)	2 (−3 – 6)	1 (−1 – 7)	0.862

Superscripts indicate significant differences between groups (a, b) after Bonferroni adjustment

*Analysis restricted to Intervention + walnut participants who consumed at least 80% of the provided walnuts

† Available sample size: control (n = 55), intervention (n = 38), intervention + walnut (n = 22)

‡ Available sample size: control (n = 46), intervention (n = 33), intervention + walnut (n = 20)

§ Available sample size: control (n = 59), intervention (n = 41), intervention + walnut (n = 23)

Significantly greater increases in energy from *fruit products and dishes* and significantly larger decreases in energy from discretionary foods/beverages were reported within the first three months for the *IW* group compared to *C* (*P *= 0.043 and *P *= 0.022, respectively) (Table 3). There were no significant differences in changes in reported food choices detected between the *IW* and *I* groups at three months, nor between *I* and *C* groups. There were no differences between groups in changes in reported consumption of key food groups at 12 months (Table 4).

There were no significant differences between groups in the reported percentage of energy from discretionary foods at baseline (*IW*: 29.7% [21.1–39.5], *I*: 27.6% [20.4–39.0], *C*: 27.4% [20.6–36.2], *P *= 0.573). At three months, all groups reported a lower percentage of energy from discretionary foods compared to baseline (*P *< 0.05),but the value was significantly lower in the *IW* group compared to the *I* or *C* (*IW*: 14.3% [9.1–22.3], *I*: 17.1% [10.7–28.6], *C*: 20.2% [12.7–29.6], *IW vs I: P *= 0.037, *IW vs C: P *= 0.001). At12 months, a significant reduction was only found for the *IW* (*P *= 0.012) and *I* (*P *= 0.001) groups, and significant differences in values were found between the *IW* and *C* groups (*IW*: 16.1% [9.8–20.5], *I*: 20.3% [14.3–27.8], *C*: 22.6 %[14.0–31.9], *IW vs C:P *= 0.012).There were no significant differences in changes in self-reported physical activity or psychological parameters between groups at three or 12 months (Tables 3 and 4).

## Discussion

When participants in this lifestyle intervention received individualized dietary advice and integrated a complimentary sample of a healthy food (30 grams of walnuts per day) in their diet they achieved greater weight loss compared to those receiving general dietary advice (Table 3). Giving dietary advice based on foods in the Australian Guide to Healthy Eating [20] appears useful, as all groups lost weight, but more specific advice translated to usual eating patterns, and even more specifically including walnuts within this pattern produces better effects. During the intensive phase of the study (baseline to three months), the inclusion of walnuts in the diet influenced diet patterns, as this group chose substantially more *fruit products and dishes*, and consumed less energy from discretionary foods and beverages (Table 3). Over time, fewer participants remained in the study. This loss of power meant that significant differences in food choices were not able to be detected at 12 months (Table 4). However, the principle of the impact of more specific dietary advice in initiating change could be observed from the study.

This result is important when considering the clinical significance of the weight change over time. The early achievements from dietary change have implications for the overall clinical management of weight loss. In this trial, for example, it may be that more intensive exercise prescription or phone coaching may have enhanced retention by providing added support for a broader commitment to lifestyle change. As provided, the advice relating to physical activity and psychological support did not result in significant differences between groups in changes in self-reported physical activity and psychological measures, although the group provided with walnuts had the largest increase in self-reported physical activity (Table 3). Mechanisms for this change in physical activity are unclear, although habitual nut consumers have previously been observed to have healthier lifestyles, including higher levels of physical activity, than non-consumers [17]. Furthermore, a previous study reported that food provision was associated with the highest appointment attendance [33], suggesting that providing study participants with a food may act as an incentive for participant engagement, which may have been the case in the present study. The preliminary results of this study suggest the need for more research exploring the effect of targeting key foods on other lifestyle parameters such as physical activity and mental wellbeing.

The weight loss observed in this study aligns with epidemiological studies indicating an inverse association between nut intake and weight gain [11–13]. Clinical trials have shown a less than expected weight gain when walnuts are added to the diet, increasing energy intake [34]. In the current study all groups followed varying levels of advice to reduce their total energy intake and for the *IW* group energy adjustments were made in prescriptions to include walnuts. There was no difference between groups in reported energy intakes at three or 12 months but the recently noted discrepancy of 20% less metabolizable energy than predicted in walnuts [14] indicates a difference in available energy. While the mechanism is unclear, research conducted in almonds suggests that the resistance of cell walls to breakdown during digestion may result in increased fecal fat excretion [35]. Increases in energy expenditure have also been reported following the consumption of peanuts [36] and almonds [37]. Where nuts are consumed instead of highly refined foods, the impact on metabolism may be twofold. In addition to the aforementioned effects generally speaking nuts are a minimally processed food and consumption of processed foods may result in lower postprandial energy expenditure than an equivalent amount of unprocessed foods [38].

The difference in weight loss between groups is also reflective of the total diet. Previous research has reported nut consumers eat more fruit, vegetables, and fish [17], and walnut consumption leads to higher diet quality [18]. During the intensive phase of our study, participants all shifted the types and amounts of foods they consumed but the walnut group achieved superior diet quality by increasing their energy intakes from *fruit products and dishes* and decreasing energy intakes from discretionary foods/beverages much more than controls. The same trend was also found when percentage energy from discretionary foods was also examined as an indicator of overall food choice behaviors. This effect, and the lack of a significant difference between the groups receiving individualized dietary advice, suggests that placing emphasis on specific foods is important in shifting dietary patterns in the desired direction. Whilst determining the precise reason for this change was outside of the scope of this analysis, it can be postulated that provision of walnuts may have facilitated consumption of other healthy foods such as fruit, for example by encouraging intake of fruit with walnuts as a snack. Likewise, trends were apparent in shifts in consumption of *vegetable products and dishes* and *fish and seafood products and dishes*, but our inability to detect significant between-group differences may reflect variability in consumption of these foods and insufficient sample size.

The inclusion of a healthy food sample such as walnuts could drive favorable dietary changes by replacing energy-dense, nutrient-poor snacks [39], but our analysis suggests the impact may go beyond snack foods. Energy-dense, nutrient-poor ‘discretionary’ foods may originate from core food groups, particularly when recipes call for large amounts of added fat and/or sugar [40]. In the current study a decrease in absolute energy intake and proportion of total energy coming from discretionary foods/beverages was seen in the walnut group. These findings, which are indicative of a change in dietary behavior, followed the pattern of weight loss observed in this study. The changes in discretionary food intake found are also particularly relevant as discretionary foods appear to contribute about 35% of the energy in the diets of Australians [41]. They are a key target for dietary change in the context of weight management.

In interpreting this research we also need to consider the significance of appropriate comparator groups in obesity research [42,43]. It has been noted that only limited treatment effects can be exposed when comparator groups involve rigorous background interventions [42,43]. In our case, the background diet was controlled for by a common reference to the foods listed in the Australian Guide to Healthy Eating, but more rigorous background control was achieved in the two groups receiving individualized dietary advice. This may explain why a significant difference in weight loss was only detected between those who received individualized advice plus walnuts (IW) compared to general advice (C) (and not between the two groups receiving individualized advice), although monitoring of reported nut consumption suggested a minimal risk of confounding from nut consumption per se.

There are further limitations to this study. We ensured our results reflected actual walnut consumption by restricting our analysis to data on those who reported consuming at least 24 grams of walnuts per day; but this reduced the sample size available for analysis which may have limited power to detect further effects. Participant drop-out and non-adherence to walnut provision over 12 months also substantially reduced the available sample size, and this reduction in statistical power may have accounted for the lack of significant changes in food intakes observed at the end of the study. The reduced sample also limits the generalizability of these results. Despite screening for nut aversions, some of the participants randomized to the walnut group chose not to consume the walnuts for various reasons. Records indicated common reasons included forgetfulness, the impact of travel, and personal preference. By 12 months this appeared to have been accentuated possibly through trial fatigue and/or the reduced intensity of intervention (down to quarterly clinic visits from monthly in the first three months). We also used self-reported methods for walnut consumption and food choice patterns, which are known to be susceptible to error, particularly in overweight and obese individuals [44]. It should also be noted that the serving size used in this study was 30 grams of walnuts. In managing weight, portion sizes are important for all foods, and our findings should be considered in the context of a daily small handful of nuts within a healthy balanced diet. Nevertheless, the provision of 30 grams of walnuts per day appeared to be well tolerated particularly during the first three months of the study, although this did appear to decrease over the duration of the study. In addition, the likelihood of confounding between groups was low, as reported usual nut intake was minimal in the other treatment groups, and congruent with a recent national survey [41].

## Conclusions

This study has demonstrated that including a core food such as walnuts in a diet plan may enhance desired dietary changes and improve weight loss. Total energy is the main dietary factor in weight loss, but analyzing the individual foods that contributes to this energy intake is highly important. The dietary impact of providing walnuts in this context presented as replacing less desirable foods with more nutrient-rich foods. In this lifestyle intervention also addressing physical activity and providing behavioral support, dietary change appeared the most potent factor in the intensive phase, but greater attention to activity and wellbeing may have enhanced retention over time.

This research has helped to expose some of the intricacies of dietary counselling by highlighting the importance of providing individualized advice, whilst also recognizing that individual foods can help build a desirable dietary pattern by association. The unique profile of walnuts and their beneficial health effects makes them a viable starting point. Future research could further explore the positioning of nutrient-rich foods within the whole diet and continue to investigate the effects of single foods on diet quality and health outcomes.
